# Genome sequencing analysis of blood cells identifies germline haplotypes strongly associated with drug resistance in osteosarcoma patients

**DOI:** 10.1186/s12885-019-5474-y

**Published:** 2019-04-16

**Authors:** Krithika Bhuvaneshwar, Michael Harris, Yuriy Gusev, Subha Madhavan, Ramaswamy Iyer, Thierry Vilboux, John Deeken, Elizabeth Yang, Sadhna Shankar

**Affiliations:** 1 Innovation Center for Biomedical Informatics, Georgetown University Medical Center, Washington DC, USA; 2 Inova Translational Medicine Institute, Fairfax, VA USA; 3 Inova Children’s Hospital, Falls Church, VA USA; 4 Center for Cancer and Blood Disorders of Northern Virginia, Pediatric Specialists of Virginia, Falls Church, VA USA; 5 George Washington University School of Medicine, Washington DC, USA; 6 Virginia Commonwealth University School of Medicine, Inova Campus, Falls Church, VA USA

**Keywords:** Whole genome sequencing, Childhood cancers, Drug resistance, Osteosarcoma, Pharmacogenomics, Genetics

## Abstract

**Background:**

Osteosarcoma is the most common malignant bone tumor in children. Survival remains poor among histologically poor responders, and there is a need to identify them at diagnosis to avoid delivering ineffective therapy. Genetic variation contributes to a wide range of response and toxicity related to chemotherapy. The aim of this study is to use sequencing of blood cells to identify germline haplotypes strongly associated with drug resistance in osteosarcoma patients.

**Methods:**

We used sequencing data from two patient datasets, from Inova Hospital and the NCI TARGET. We explored the effect of mutation hotspots, in the form of haplotypes, associated with relapse outcome. We then mapped the single nucleotide polymorphisms (SNPs) in these haplotypes to genes and pathways. We also performed a targeted analysis of mutations in Drug Metabolizing Enzymes and Transporter (DMET) genes associated with tumor necrosis and survival.

**Results:**

We found intronic and intergenic hotspot regions from 26 genes common to both the TARGET and INOVA datasets significantly associated with relapse outcome. Among significant results were mutations in genes belonging to AKR enzyme family, cell-cell adhesion biological process and the PI3K pathways; as well as variants in SLC22 family associated with both tumor necrosis and overall survival. The SNPs from our results were confirmed using Sanger sequencing. Our results included known as well as novel SNPs and haplotypes in genes associated with drug resistance.

**Conclusion:**

We show that combining next generation sequencing data from multiple datasets and defined clinical data can better identify relevant pathway associations and clinically actionable variants, as well as provide insights into drug response mechanisms.

**Electronic supplementary material:**

The online version of this article (10.1186/s12885-019-5474-y) contains supplementary material, which is available to authorized users.

## Background

Osteosarcoma (OS) is the commonest malignant bone tumor in children. It accounts for about 2% of childhood cancers in the US. Approximately 800 new cases are diagnosed in the US every year, about 400 of which are in children and teens [1]. OS occurs mostly between the ages of 10 and 30. Approximately 60% of all malignant bone tumors diagnosed in the first two decades of life are osteosarcomas.

The role of adjuvant chemotherapy is well established in the treatment of osteosarcoma [2, 3]. Prior to use of systemic chemotherapy, two-year survival was less than 20% even in patients with clinically localized disease. Most recent studies report a 3-year survival of 60–70% among patients with non-metastatic disease treated with combination chemotherapy. Standard treatment for non-metastatic osteosarcoma includes neo-adjuvant chemotherapy followed by surgical resection and post-operative chemotherapy. The extent of necrosis of the primary tumor at time of definitive surgical resection is the only significant prognostic factor in patients with non-metastatic osteosarcoma. Patients with less than 10% viable tumor at time of definitive surgery have significantly lower risk of relapse as compared to those with more than 10% viable tumor [4]. Five-year survival is 75–80% among patients with good histological response and only 45–55% among poor responders even after complete surgical resection [5, 6].

The most active chemotherapeutic agents against osteosarcoma include cisplatin, doxorubicin and high dose methotrexate. The Children’s Oncology Group (COG) considers combination cisplatin, doxorubicin and high dose methotrexate (MAP) as standard therapy for osteosarcoma [7]. Postoperative therapy is often modified in poor responders to improve outcome, such as the use of ifosfamide and etoposide [8–10]. However, no such attempts have been successful to date. It is likely that the initial 8–12 weeks of ineffective therapy select for resistant clones and allow the cancer cells to metastasize. Changing therapy at a later time point fails to change the outcome. There is a need to identify the poor responders at time of initial diagnosis to avoid delivering ineffective pre-operative therapy. Alternative chemotherapeutic agents at initial diagnosis could potentially alter outcomes in patients expected to have poor response to standard cisplatin based chemotherapy. Thus, the key challenge is to determine the basis for response and non-response in patients at the outset and identify patients who are eligible for intensified or alternative therapy given their personal profile.

The completion of the HapMap project is a historic achievement [11]. The project identified over one million single nucleotide polymorphisms (SNPs) across the human genome, which may be at the root of the great variation seen in human health and disease. Germline genetic variation between individuals may lie at the heart of two critical questions: who is at risk to develop cancer, and how best to treat individuals once they are diagnosed. This genetic variation may account for the wide variation seen in the response and toxicity related to chemotherapeutic agents.

The pharmacogenetic differences between patients are multi-factorial [12]. One factor is polymorphism in drug targets, including cell surface receptors and target proteins. Another is polymorphism in cellular recovery mechanisms that repair cytotoxic agent-induced damage. Finally, there are polymorphisms in genes encoding proteins involved in drug pharmacokinetics, including proteins that impact drug absorption, metabolism, distribution, and elimination (ADME). Germline pharmacogenetic biomarkers have been found for a number of anticancer agents, including irinotecan, mercaptopurine, 5-flurouracil, and tamoxifen [13–16]. These studies often used a candidate gene approach, and attempted to explain a drug’s efficacy or toxicity by identifying one gene and even one variant within that gene [17].

While candidate gene/single variant analysis provides important insights, there are several limitations in most published studies. The implicated alleles were often low frequency and the absolute numbers of patients with these alleles were low. Only one or few single nucleotide polymorphisms (SNPs) were examined for each gene of interest and potentially significant polymorphisms could have been missed. In this study, we applied both single SNP and multiple SNP analysis to get an enhanced understanding of genetic polymorphism in the disease.

A genome-wide approach enables examination of polymorphisms of a large number of implicated genes in multiple pathways that may impact on response to chemotherapy. With advance in technology and reduction in costs, whole-genome sequencing is now feasible with next-generation sequencing. Complete sequences of implicated genes can be analyzed to identify polymorphisms in the context of pathway datasets, rather than as individual data points. An integrative approach combining whole genome sequencing (WGS) with multiple datasets and defined clinical data can 1) better capture pathway associations, and 2) provide the opportunity for discovery of clinically actionable variants [18, 19].

We have previously used this integrative approach to define the pharmacogenetic profile of gemcitabine, and defined a ‘sensitive’ and ‘resistant’ genotype using the combination of pre-clinical data from the NCI60 cell lines and a genome wide association studies (GWAS) clinical dataset from a large clinical trial [20]. We now wish to expand this approach to pediatric osteosarcoma patients treated with multi-agent chemotherapy, including cisplatin, doxorubicin, and methotrexate.

The aim of this study is to identify, test, and validate a genotype resistant to cisplatin, doxorubicin, and methotrexate, in children with osteosarcoma, using two datasets derived from clinical samples. The genetic signature that is strongly associated with drug response could be translated into clinical oncology [21] and used in the future to personalize therapy.

## Methods

Whole genome sequencing data was obtained from a cohort of 15 osteosarcoma patients from the Inova Fairfax Hospital for Children. Additional sequencing data and clinical outcomes of 85 patients with osteosarcoma were obtained from the NCI TARGET dataset. The following sections describe the datasets, sample collection procedures and data analyses.

### Datasets

- Inova Pediatric Group Osteosarcoma Patients (labeled ‘INOVA’): Whole genome sequencing (WGS) was performed for 15 children, who are up to 21 years of age with osteosarcoma, including newly diagnosed and off therapy. Patients were recruited over a 2 year period from 2014 to 2016. Demographic, clinical outcome, chemotherapeutic exposure and pathological response data were collected for all subjects. The study was IRB approved; informed consent and assent were obtained from each child and parent as appropriate. DNA was extracted from whole blood samples, and whole genomic DNA was sequenced on an Illumina HiSeq 2500 whole genome sequencer at 30-50X coverage. Raw sequencing data were obtained in the form of Fastq [22] files.
- TARGET Osteosarcoma Dataset (labeled ‘TARGET’): This is a cohort of 85 fully characterized patient cases from NCI’s TARGET database for osteosarcoma (released in Feb 2015) [23, 24]. The majority of these patients were teenagers. The samples were collected at the time of diagnostic surgery. Aligned genomic data from whole blood samples were downloaded from NCI’s dbGAP database [25]. Out of this 85-patient cohort, whole exome sequencing (WXS) data were available on 52 patients and WGS data were available on 33 patients. For each patient, aligned genomic data in the form of ‘BAM files’ short for Binary Alignment Map [26] were downloaded, decrypted and processed.

All patients were treated with standard MAP therapy. The standard MAP neo-adjuvant chemotherapy regimen is as follows: Doxorubicin (Adriamycin) 37.5 mg/m2/day and Cisplatin (Platinum) 60 mg/m2/day on days 1,2 of week 1; Methotrexate 12 g/m2 on day 1 of week 4, week 5. This entire cycle is repeated week 6–10. Surgical resection with tumor necrosis data on week 12.

### Processing of genomic data

We used open source well-known best practices tools in the processing of sequencing data. The tools included Sickle [27], Bowtie2 [28], Samtools [29], Picard [30], and GATK’s [31] HaplotypeCaller. After quality control, the raw sequencing data in the form of FASTQ files were aligned to the human reference genome (version hg19). Post alignment processing was done on the aligned reads, so that it will be in the right format for the subsequent step. A variant calling algorithm was applied, which mathematically checked the patient genome against the reference genome to identify variants in the form of single nucleotide polymorphisms (SNPs) or indels. The variants identified from each patient were merged into one file. Only those variants that passed quality check were chosen for further analysis. Additional file 1 shows the steps, file formats and tools in this genomic data processing, which was performed on an Amazon cloud r3.4xlarge instance.

The TARGET variants were a combination of whole-exome and whole-genome data. We applied a filtering criterion on the variants such that if all patients, or if 84 of the 85 patients had the reference allele, then the variant was rejected. The same processing steps were applied to both datasets in an effort to reduce batch effects. At the end of this filtering, the TARGET dataset had about 900,000 SNPs and the INOVA dataset had about 8 million SNPs.

### Outcomes of interest

The outcomes of interest chosen were: (1) Relapse (2) Percent tumor necrosis and (3) overall survival.

Tumor necrosis following preoperative chemotherapy is the strongest prognostic factor for osteosarcoma [32]. Tumor necrosis as an outcome provides a window into the early part of drug response, while ‘relapse’ as outcome provides an extended view of drug response.

Relapse information was available for all the 15 INOVA patients and 85 TARGET patients. These patients also had overall survival information (Table 1).

**Table 1:** Summary of patients showing the number of patients who relapsed and were relapse-free

Dataset	# Relapse	# Relapse-free	Total # of patients
TARGET	39	46	85
INOVA	3	12	15

Tumor necrosis data were available for the 15 patients from the INOVA cohort, but only for 44 out of 85 patients from the TARGET cohort. Patients with tumor necrosis greater than 90% at the time of resection were ‘Good responders’; patients with tumor necrosis <= 90% were ‘Poor responders’ (Table 2).

**Table 2:** Summary of patients with Tumor necrosis information. Good Responders: > 90% tumor necrosis; Poor Responders: ≤ 90% tumor necrosis

Dataset	# Poor Responders	# Good Responders	Total # of patients
TARGET	26	18	44
INOVA	9	6	15

## Methods for SNP analysis

**Fig. 1:**
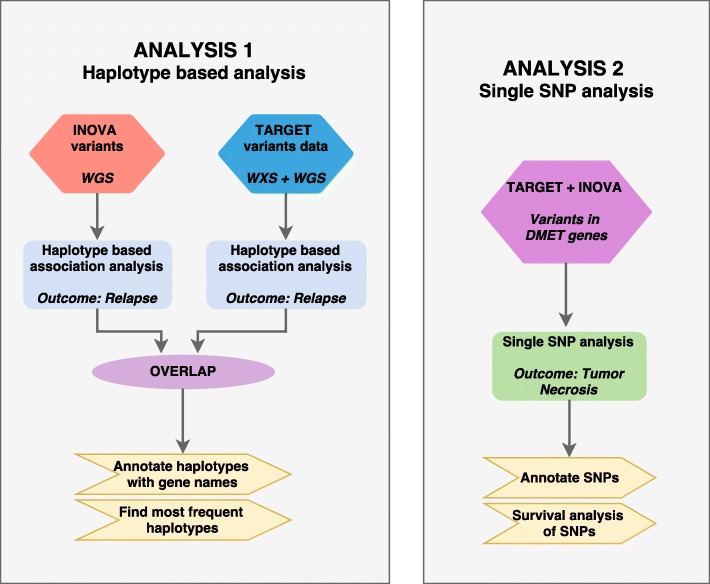
Schematic of analysis methodology

Two analyses were performed, shown in Fig. 1.

### Analysis 1: hotspots associated with relapse

We analyzed the INOVA and TARGET datasets separately to look for hotspots associated with relapse outcome. Hotspots are regions in the genome that have multiple co-occurring mutations in the same or close-by regions [33]. In our analysis, we looked for hotspots, in terms of haplotypes, which are groups of markers (SNPs) that are inherited together [34] . Once the significant haplotypes associated with outcome were identified, we then looked for haplotypes overlapping between the two datasets.

There are several advantages to grouping SNPs: it reduces the number of tests, making it easier to reject the null hypothesis, offering more power to the analysis; SNPs in a haplotype block (haploblock) are inherited together; the markers (SNPs) are often closely located and will be in linkage disequilibrium (LD); increased robustness in statistical testing; groups of SNPs affecting outcome are more biologically relevant than a single SNP affecting outcome [35].

For this study, we performed haplotype based association test analysis using the PLINK tool [36, 37]. For each dataset, the tool identified haplotypes significantly associated with relapse outcome (*p* value <= 0.05). Using chromosome location as criteria, we looked for overlapping haplotypes between the two datasets. We looked for partial or complete overlap in the chromosome location; hence the SNPs in these common haplotypes may or may not be the same. Looking for overlap in two independent datasets reduces the chances of randomly occurring haplotypes and helps eliminate false positives.

The SNPs in these common haplotypes were then mapped to genes and pathways to enable downstream system biology analysis to give insights into drug response mechanisms.

### Analysis 2: targeted analysis of variants in DMET genes associated with tumor necrosis and survival

We performed a targeted analysis of mutations in the genes involved in drug absorption, metabolism, distribution, and elimination (ADME), also known as Drug Metabolizing Enzymes and Transporters (DMET). The words DMET and ADME are used interchangeably in this manuscript. We explored their association with tumor necrosis using machine learning methods.

Among the 85 TARGET patients, only 44 had clinical data available on tumor necrosis (Table 1). All the 15 INOVA patients had data available on tumor necrosis. Since the sample size would be small and there would not be enough power to obtain statistically significant results, we merged the two datasets to get a total of 59 patients (referred to as the ‘TARGET+INOVA’ cohort).

Before starting the analysis, we tested the data for batch effects. We performed a principal component analysis (PCA) on variants in DMET genes (Additional file 2) using the R statistical platform (https://cran.r-project.org/). We found a clear batch effect due to the merging of the two datasets. We have accounted for this batch effect in our analysis.

From the TARGET + INOVA cohort, we extracted 36,504 variants in DMET genes. We binned the data into 0 (indicating no mutation) and 1 (indicating presence of mutation). 85% of these data were randomly set as training set and the rest 15% were set as an independent validation set using the caret package [38] (seed of 7) in R. Several filters were applied on the training set so that only SNPs with the most variability were chosen for the analysis. At the end of all the filtering, we were left with 4543 SNPs for analysis.

For each of the 4543 variants, we created two generalized linear models (GLMs) with tumor necrosis as outcome variable: one with the variant and adjusting for the dataset to control for batch effect; and second one specified without the variant. This allowed us to perform logistic regression analysis to find those variants most associated with tumor necrosis outcome. We then used analysis of variance (ANOVA) to compare the two models to obtain a *p*-value based on chi-square distribution (described in Additional file 2). The *p*-values from this test were adjusted for multiple testing using the Benjamini Hochberg approach to control the false discovery rate (FDR) [39]. The significant variants (*p* value < 0.05) from this analysis were annotated using SnpEff [40] variant annotation tool. The annotations were used to sub-divide these SNPs based on their impact into high, moderate, low impact, and modifiers (non-coding regions).

We built a Random Forest predictive model with these significant variants with 20 fold cross validation (seed 260), and performed a prediction on the independent validation set (block diagram shown in Additional file 2).

We also performed survival analysis on the significant variants to assess their impact on overall survival. The association with survival was tested using the log rank test statistic [41]. The analysis was performed in the R programming language. Kaplan Meier survival curves [42] were generated. Results were compared to published literature.

### Validation of SNPs detected using sanger sequencing

The significant SNPs and indels obtained from our analysis of WGS data were confirmed in the lab using Sanger Sequencing technique. DNA extracted from the INOVA cohort was used for PCR amplification. Using Primer3 (http://bioinfo.ut.ee/primer3-0.4.0/), primers were designed to cover and validate the subset of variants identified by WGS. The PCR was performed using AmpliTaq Gold 360 master mix (Applied Biosystems). After Exo/SAP purification (ThermoFisher Scientific), the amplicons were sequenced by using the BigDye V.3.1 Terminator chemistry (Applied Biosystems) and separated on an ABI 3730xl genetic analyzer (Applied Biosystems). Data were evaluated using Sequencher V.5.0 software (Gene Codes).

This validation was performed on three of our analysis results: (a) variants belonging to the most frequent haplotypes in the INOVA dataset (b) variants in the form of dbSNP ids amongst the overlapping haplotypes associated with relapse and common to the two datasets (c) variants among the DMET genes significantly associated with tumor necrosis and overall survival.

## Results

### Mutation hotspots associated with relapse

We found a total of 2178 haplotypes significantly associated (*p* value < 0.05) with relapse outcome in the TARGET dataset and 110,000 significant haplotypes in the INOVA dataset (more haplotypes identified in INOVA data because it was WGS data). Using chromosome location as the criteria for finding overlapping haplotypes between the two datasets, we found a total of 231 overlapping haplotypes (Additional file 3). We mapped the SNPs in these haplotypes to genes, and found 26 genes common to the TARGET and INOVA datasets associated with relapse, including AKR1D1, SLC13A2, MKI67 and PIK3R1 and others (Table 3).

**Table 3:** List of 26 common genes obtained from haplotypes overlapping between INOVA and TARGET and associated with relapse outcome

List of 26 common genes	
7SK	
AKR1D1	
C10orf112 (also known as MALDR1) ^*^	
CACNA2D4	
CDH13^$^	
CDH9^$^	
CDRT15	
CSMD1	
DGCR6^*^	
DQ576041	
DQ600701 (also known as PIR61811) ^*^	
DQ786190	
GABRG3^$^	
HBE1^$^	
LOC643401	
MKI67	
OCA2	
OR51B5	
PCGF2	
PDZD4^*^	
PIK3R1^*$^	
PKHD1	
PPP1R12C^*^	
SLC13A2^*$^	
ZNF321P	
ZNF816	

Genes marked with * indicate the most frequent haplotypes associated with relapse

Genes marked with ^$^ were significantly enriched in the pathway enrichment analysis (details in Table 4)

We also performed enrichment analysis using Reactome database (https://reactome.org/) [43] to map these 26 genes to pathways (Table 4).

**Table 4:** Pathways enriched from the 26 genes common to TARGET and INOVA haplotypes

Pathway name	#Entities found	#Entities total	Entities *P* value	Entities FDR	Submitted entities found
Adherens junctions interactions	2	35	0.003	0.228	CDH13; CDH9
Cell-Cell communication	3	133	0.003	0.228	CDH13; PIK3R1; CDH9
Factors involved in megakaryocyte development and platelet production	3	179	0.007	0.228	HBE1
Cell-cell junction organization	2	67	0.009	0.228	CDH13; CDH9
Cell junction organization	2	94	0.017	0.228	CDH13; CDH9
Sodium-coupled sulphate, di- and tri-carboxylate transporters	1	9	0.019	0.228	SLC13A2
MET activates PI3K/AKT signaling	1	10	0.021	0.228	PIK3R1
GP1b-IX-V activation signaling	1	12	0.025	0.228	PIK3R1
PI3K events in ERBB4 signaling	1	15	0.032	0.228	PIK3R1
GABA A receptor activation	1	15	0.032	0.228	GABRG3
Signaling by FGFR3 fusions in cancer	1	16	0.034	0.228	PIK3R1
Erythrocytes take up oxygen and release carbon dioxide	1	16	0.034	0.228	HBE1
Signaling by FGFR4 in disease	1	18	0.038	0.228	PIK3R1
PI3K events in ERBB2 signaling	1	22	0.046	0.228	PIK3R1
Tie2 Signaling	1	22	0.046	0.228	PIK3R1

### Most frequent haplotypes amongst the overlapping hotspots

Among the 231 overlapping haplotypes between the INOVA and TARGET datasets, we looked in detail at those haplotypes that have the highest sample frequency in each dataset (Table 5).

**Table 5:** Most frequent haplotypes in the TARGET and INOVA datasets

		TARGET _NSNP	TARGENHAP	TARGET_CHR	TARGET_BP1	TARGET_BP2	TARGET_Haplo	TARGET_Region	TARGET_Genes	TARGET_F		INOVA_NSNP	INOVA_NHAP	INOVA_CHR	INOVA_BP1	INOVA_BP2	INOVA_Haplo	Inova_Region	Inova_Genes	Inova_F
Haplotype#1	Most frequent in Target	3	2	23	153,056,311	153,084,802	222	intronic, exonic	IDH3G, PDZD4	0.699		5	5	23	153,073,319	153,075,655	22,222	intronic	PDZD4	0.167
Haplotype#4	Most frequent in Target	8	5	22	18,878,593	18,879,911	22,222,122	intergenic	DQ786190, DGCR6	0.506		8	8	22	18,877,869	18,878,859	22,122,121	intergenic	DQ786190, DGCR6	0.106
Haplotype#12	Most frequent in Target	7	6	22	18,878,593	18,879,898	2,222,212	intergenic	DQ786190, DGCR6	0.487		8	8	22	18,877,869	18,878,859	22,122,121	intergenic	DQ786190, DGCR6	0.106
Haplotype#20	Most frequent in Target	8	4	22	18,878,349	18,878,632	11,222,221	intergenic	DQ786190, DGCR6	0.478		8	8	22	18,877,869	18,878,859	22,122,121	intergenic	DQ786190, DGCR6	0.106
Haplotype#31	Most frequent in Target	2	3	17	26,816,365	26,817,537	11	intronic, exonic	SLC13A2	0.353		8	9	17	26,816,365	26,822,518	11,121,221	intronic, exonic	SLC13A2	0.0839
Haplotype#32	Most frequent in Target	3	4	17	26,816,365	26,818,676	112	intronic, exonic	SLC13A2	0.341		8	9	17	26,816,365	26,822,518	11,121,221	intronic, exonic	SLC13A2	0.0839
Haplotype#61	Most frequent in Inova	3	6	19	55,614,923	55,624,113	111	intronic, exonic	PPP1R12C	0.125		7	7	19	55,619,303	55,622,518	2,222,222	intronic	PPP1R12C	0.71
Haplotype#74	Most frequent in Inova	3	6	19	55,614,923	55,624,113	111	intronic, exonic	PPP1R12C	0.125		8	8	19	55,618,992	55,622,518	22,222,222	intronic	PPP1R12C	0.675
Haplotype#126	Most frequent in Inova	5	16	5	67,513,481	67,554,172	11,121	intronic	PIK3R1	0.0625		5	9	5	67,548,442	67,549,836	22,222	intronic	PIK3R1	0.448
Haplotype#160	Most frequent in Inova	7	12	10	19,620,439	19,641,239	1,221,212	intergenic	DQ600701, C10orf112 (MALRD1)	0.0409		7	9	10	19,621,103	19,624,121	2,222,221	intergenic	DQ600701, C10orf112 (MALRD1)	0.267

NSNP: Number of SNPs in the haplotype, NHAP: Number of common haplotypes, CHR: Chromosome, BP1: Position of left most SNP, BP2: Position of right most SNP, Haplo: Indicates the haplotype that was formed. The numbers 1 and 2 represent the genotypes. F: Sample frequency

### Common SNPs amongst the overlapping hotspots

Using dbSNP ids as criteria, we looked for common SNPs amongst the 231 overlapping hotspots (haplotypes) between TARGET and INOVA datasets. We found 10 dbSNP ids common to the two datasets. These include four SNPs in MKI67 (rs7071768, rs11016073, rs61738284, rs11591817), one SNP in CACNA2D4 (rs10735005), three SNPs in SLC13A2 (rs3217046, rs11568466, rs9890678), and two SNPs in PPP1R12C (rs10573756, rs34521018). These variants are important because they are part of hotspots that are significantly associated with relapse outcome. The fact that these same SNPs were found as part of hotspots in two independent datasets, and validated in the lab, indicates their potential as biomarkers for diagnostics and drug discovery.

### Targeted analysis of variants in DMET genes associated with tumor necrosis and survival

We explored the association of variants in DMET genes (referred to as ‘DMET variants’) with tumor necrosis as outcome, and obtained a total of 281 variants with *p* value < 0.05 that can separate good responders and poor responders (Additional file 4). We built a predictive model using these variants. This model gave us a prediction accuracy of 87.5% when applied to the independent validation set. The confusion matrix and the summary of the model are shown in Additional file 5. We annotated these variants with SnpEff variant annotation tool [40] and grouped them into high impact, moderate impact, and modifier (non-coding regions).

A “high impact” variant is one that is predicted to have a disruptive impact in the protein, such as protein truncation, loss of function or triggering nonsense mediated decay. A “moderate impact” variant is a non-disruptive variant that might change protein effectiveness. A “low impact” variant is assumed to be harmless or unlikely to change protein behavior. “Modifier variants” are non-coding variants or variants affecting non-coding genes [40].

Out of the 281 variants significantly associated with tumor necrosis, there was one high impact variant, rs17143187, which is a splice donor variant in the ABCB5 gene. This variant is located on the boundary of exon and intron and could cause aberrant splicing that would result in a disrupted protein [44]. Sixteen moderate impact variants, 15 low impact variants, and 249 modifier variants were identified. 210 out of the 281 of these variants were located in intronic regions.

**Fig. 2:**
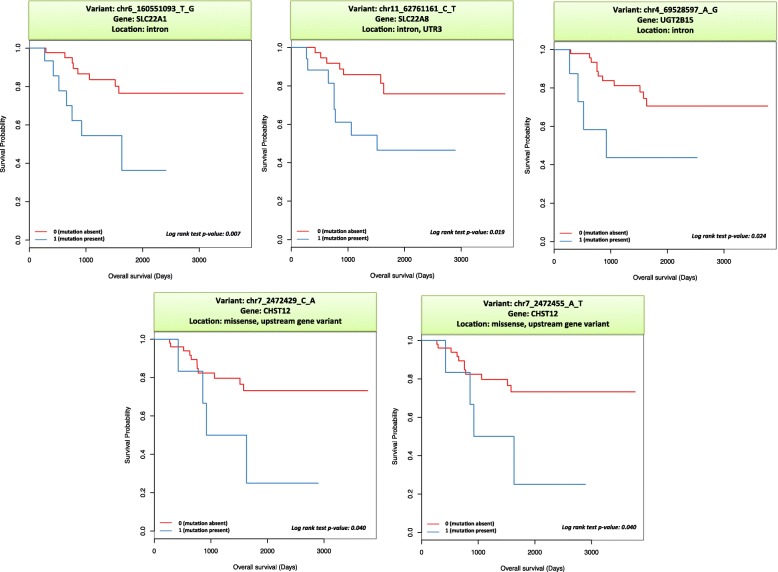
Kaplan Meier survival curves for the significant mutations in DMET genes. 0 (red): no mutation, and 1 (blue): presence of mutation

We performed survival analysis on these 281 variants, and obtained five variants as significant results (*p* value < 0.05). Thus, these 5 variants are significantly associated with both tumor necrosis and overall survival (Table 6). The Kaplan Meier survival curves for these five variants are shown in Fig. 2.

**Table 6:** Results of survival analysis showing association of variations with overall survival

Name Of Variant	# of Samples without mutation	# of samples with mutation	# of events in samples without mutation	# of events in samples with mutation	*P* value from Log Rank (SC) Test	Adjusted p-value	Hazard Ratio*	Gene Name	ENCODE annotation	dbSNP id	Impact	Location
chr6_160551093_T_G	44	15	8	7	0.007	0.59	3.723	SLC22A1	Heterochromatin; low signal	rs4646272	Modifier	Intron variant
chr11_62761161_C_T	42	17	7	8	0.019	0.59	3.161	SLC22A8	Repressed	rs2187384	Modifier	Intron variant, UTR3
chr4_69528597_A_G	51	8	11	4	0.024	0.59	3.462	UGT2B15	.	rs34073924	Modifier	Intron variant
chr7_2472429_C_A	53	6	11	4	0.040	0.59	3.124	CHST12	Transcription Elongation	rs3735099	Moderate, modifier	Missense variant; upstream gene variant
chr7_2472455_A_T	53	6	11	4	0.040	0.59	3.124	CHST12	Transcription Elongation	rs3735100	Moderate, modifier	Missense variant; upstream gene variant

Variant name is defined as “ChromsomeNumber_Position_ReferenceAllele_AlternateAllele”

The Hazard ratio was obtained from the Cox Proportional Hazards Test – indicates hazard value of having an event (death) in the mutation group compared to the non-mutation group

### Validation of SNPs detected using sanger sequencing

The lab validation experiment was performed on three groups of analysis results: (a) 20 variants belonging to the most frequent haplotypes in the INOVA dataset (labeled as ‘Group A’) (b) 10 variants in the form of dbSNP ids common to the two datasets amongst the overlapping haplotypes significantly associated with relapse (labeled as ‘Group B’) and (c) 5 variants among the DMET genes significantly associated with tumor necrosis and overall survival (labeled as ‘Group C’).

Among the variants in Group A, at-least one SNP in each haplotype was successfully validated (Additional file 6). Validation of seven variants (located in intronic regions of two genes and one intergenic region) was not confirmed. Several of those that were not confirmed were located in insertion/deletion regions. All the variants in Groups B and C were successfully validated (Additional file 6).

## Discussion

### Summary of results

The main results are summarized in Table 7, showing (a) the 26 genes from common haplotypes, found in both the TARGET and INOVA datasets, that are associated with relapse; and (b) The 10 dbSNP ids among the overlapping hotspot regions common to the two datasets and (c) the genes from targeted DMET analysis associated with both tumor necrosis and overall survival. We explored these genes to see which of them are known in relation to drug response from published literature.

**Table 7:** Summary of results obtained at the gene level

(a) List of 26 common genes from overlapping haplotypes associated with relapse	(b) List of 10 dbSNP ids common to the two datasets amongst the overlapping haplotypes associated with relapse	(c) Genes from targeted DMET analysis associated with both tumor necrosis and overall survival
7SK	rs7071768 (MKI67)	SLC22A8
AKR1D1	rs11016073 (MKI67)	SLC22A1
CACNA2D4	rs61738284 (MKI67)	UGT2B15
CDH13	rs11591817 (MKI67)	CHST12
CDH9	rs10735005 (CACNA2D4)	
CDRT15	rs3217046 (SLC13A2)	
CSMD1	rs11568466 (SLC13A2)	
DGCR6*	rs9890678 (SLC13A2)	
DQ576041	rs10573756 (PPP1R12C)	
DQ600701 (also known as PIR61811) *	rs34521018 (PPP1R12C)	
DQ786190		
GABRG3		
HBE1		
LOC643401		
MALDR1 (also known as C10orf112) *		
MKI67		
OCA2		
OR51B5		
PCGF2		
PDZD4 *		
PIK3R1 *		
PKHD1		
PPP1R12C*		
SLC13A2*		
ZNF321P		
ZNF816		

Genes marked with * indicate the most frequent haplotypes associated with relapse

### Hotspots associated with relapse

We performed haplotype based association analysis in the TARGET and INOVA datasets to find haplotypes associated with relapse outcome, and then looked for overlap. We found 231 haplotypes overlapping (based on chromosome location) amongst the TARGET and INOVA datasets. The SNPs in these haplotypes were mapped to genes, and 26 genes were found common between the two datasets. These 26 common genes were explored for known relationships with drug response.

Among them is AKR1D1, which has SNPs rs1872929 and rs1872930 among the hotspots in the TARGET dataset, and are in the three prime untranslated region (3′ UTR) of AKR1D1. SNPs rs1872929 and rs1872930 were found as part of a haplotype and in LD with intronic SNP rs2306847 (found among the hotpots in INOVA dataset) as part of a haplotype [45]. These SNPs have been significantly associated with higher AKR1D1 mRNA expression [45]. AKR1D1 is a key genetic regulator of the P450 network, which affects drug metabolism, efficacy and adverse events in patients [45].

Genes CDH13, and CDH9 are part of the cadherin family of genes, and along with PKHD1, are part of the cell-cell adhesion biological process. This biological process is associated with a multidrug resistant phenotype, “cell adhesion-mediated drug resistance,” or CAM-DR [46]. Osteoblasts express multiple cadherins [47], and cadherin mediated cell-to-cell adhesion is critical for normal human osteoblast differentiation [47]. The cadherin family of genes is associated with CCN3, which has been found to have prognostic value in Osteosarcoma [48]. CDH13 and CHD9 are also part of the adherens junction biological process; and adherens-dependent PI3K/AKT activation is known to induce resistance to genotoxin-induced cell death in intestinal epithelial cells [49].

Variants in gene ZNF321P (among the TARGET haplotypes) and in gene PCGF2 (among the INOVA haplotypes) are intronic and also located in active promoter regions. Similarly, intronic variants in gene PPP1R12C (among the INOVA haplotypes) are located in strong enhancer regions containing transcription factor binding sites. Mutations in such gene regulatory regions could inhibit transcription factor binding, leading to aberrant cell proliferation or drug response [50].

We hence see that some of the genes identified from our analysis are linked with drug resistance or drug response; others have not been previously linked with drug response. In the genes previously linked with drug resistance or response (AKR1D1, CDH13, and CDH9), the SNPs and haplotypes found from our analyses are novel.

### Most frequent haplotypes

Among the 231 common haplotypes between the INOVA and TARGET datasets, we examined the haplotypes that have the highest sample frequency (Table 5). The most frequent haplotype in the TARGET dataset span intronic and intergenic regions in or near the following genes: IDH3G, PDZD4, DQ786190, DGCR6, and SLC13A2. The most frequent haplotypes in the INOVA dataset span the region in or near the following genes: PPP1R12C, PIK3R1, DQ600701 and MALRD1.

DQ600701 (also known as PIR61811) is a Piwi-interacting RNA (piRNA), a small non-coding RNA found in clusters as regulatory elements, and control gene expression in germ cells [51–53] .Somatic cells express similar small non-coding RNAs called piRNA-like (piR-Ls or pilRNA) with similar functions as piRNAs. piRNA/pilRNAs appear to target the 3′ UTR of mRNAs and potentially regulate mRNA translation [53–55] and possibly affect drug response. For example, pilRNAs were found to play key roles in chemo resistance to cisplatin-based chemotherapy in lung squamous cell carcinoma (LSCC) [56].

Another frequent haplotype is located in the intergenic region between DQ786190 and DGCR6 [57, 58], which is located in chr 22, q11.21 region. According to Genbank, the mRNA sequence DQ786190 is involved in lineage-specific gene duplication and loss in humans [59]. According to ENCODE annotation, this intergenic region with chromosome position 18,878,593–18,878,632 contains repetitive/copy number mutations. The INOVA dataset also contains nearby haplotypes (position 18,877,874–18,878,489), which spans the intergenic region between DQ786190 and DGCR6. This region contains TATA boxes, therefore haplotypes containing multiple SNPs in this intergenic region can potentially affect transcription or gene copy number, and possibly drug response [60]. miR-145 is predicted to target this DQ786190 mRNA sequence [40]. miR-145 was found to be 5 times under expressed in the miRNA expression signature associated with canine osteosarcoma [61]. Thus, the variant haplotype could affect drug response through decreased miRNA binding [62].

SLC13A2 is the only Drug Metabolizing Enzyme and Transporter (DMET) gene that has significant haplotypes present in the TARGET and the INOVA dataset. This gene is known to play an important role in transporter activity [63]. SLC13A2 was down regulated along with miR-9 overexpression in malignant murine mastocytoma cell lines, and in primary canine osteosarcoma (OSA) tumors and cell lines [64]. Another gene in the same solute carrier family is SLC19A1, which is a folate carrier. Reduced folate carrier function has been associated with impaired methotrexate transport in osteosarcoma tumors [65, 66]. Polymorphisms in SLC19A1 have been associated with response to methotrexate treatment in pediatric osteosarcoma [67, 68]. In recent years, these SLC transporters have been recognized as having the potential to transport and deliver anticancer chemotherapeutic agents, and are being studied as drug targets in cancer [69, 70].

Another gene in the list is PIK3R1, which encodes regulatory subunits of PI3-kinase [71]. The PI3K pathway is frequently activated in cancer due to genetic (e.g., amplifications, mutations, deletions) and epigenetic (e.g., methylation, regulation by non-coding RNAs) aberrations targeting its key components, and may affect response to specific therapeutic agents [72]. Hotspots in exonic regions of PIK3R1 (residue M582_splice, N564, G376, R348, K567) have been found in tumor samples of various cancers (http://cancerhotspots.org/) [73]. In Zhao et al., the authors found that up-regulation of long non-coding RNA promoted osteosarcoma proliferation and migration through the regulation of PIK3IP1, another protein in the PI3K pathway [74].

### Common SNPs amongst the overlapping hotspots

The 4 SNPs in the MKI67 gene rs7071768, rs11016073, rs61738284 and rs11591817 are present in the hotspots in the TARGET and INOVA datasets. All these SNPs are non-synonymous and are expected to affect protein function. According to ENCODE annotation, these SNPs are located in regions of transcription elongation, which can have broad effects on gene expression. Mutations in these regulatory regions have been linked with disease mechanisms [75] and possibly modified drug response. The gene MKI67 is often used as a surrogate biomarker to score the aggressiveness of the tumor, and expression of this gene has been used as a predictor of response to chemotherapy in breast cancer patients [76, 77].

### Targeted analysis of variants in DMET genes associated with tumor necrosis and overall survival

Among the 281 DMET variants that were significantly associated with tumor necrosis, was gene ABCG2. This gene is part of the Methotrexate metabolic pathway (MTX pathway), and mutations in this gene have been implicated in MTX efficacy and toxicity. This is gene is also linked with breast cancer treatment resistance [78].

The only high impact variant was rs17143187, which is a splicing and intron variant in the ABCB5 gene. Alternative splicing and ABCB5 SNPs are known to affect drug response [79–81]. This mutation is predicted to be a deleterious mutation based on FATHMM prediction algorithm [82].

Among the 281 variants that were significantly associated with tumor necrosis, 5 variants were significantly associated with overall survival as well. All these 5 variants have a hazard ratio of 3, meaning that at any particular time, three times as many patients in the mutation group are experiencing an event (death) compared to patients in the non-mutation group.

A total of 210 of the 281 significant variants were located in intronic regions, which is consistent with known literature. Luizon and Ahituv reviewed all published pharmacogenomics genome wide association studies (GWAS) and found that 96.4% of the SNPs reside in noncoding regions [50]. Intronic regions typically harbor microRNAs and long non-coding RNA, other regulatory elements, epigenetic elements and structural variants [83, 84]. These intronic regions are sites of intron retention, in which the introns are not spliced out, but are retained. Recent studies have shown that intron retention affects regulation of gene expression and RNA translation [85, 86].

Variants in introns can affect drug response by altering the gene expression [83, 87–91]. In recent years, noncoding RNA are being researched as potential drug targets since they affect gene expression and disease progression [92]. Enhancers have been identified as potential biomarkers for early cancer detection, and targets for cancer therapy [93]. Hotspot regions are being researched for their potential as targets for diagnosis and drug development [73, 94].

Hence, some of the significant variants obtained from our analyses are supported by reports in the literature and serve as in-silico validation of our results, while other variants are novel, and offer additional value for exploration of new and novel drug therapies.

### Limitations

Although the major findings were statistically significant and confirmed by Sanger sequencing, the sample size of the INOVA cohort was relatively low. The relevance of these findings with respect to drug response needs to be validated in a larger independent cohort of patients before applying in clinics. As next steps, we plan to explore the application of the significant haplotypes and single SNPs discovered, in a larger scale study to build a predictive signature of genomic variants associated with treatment outcome.

A recent publication on pan-can analyses of pediatric cancers by the TARGET group [24] published in March 2018, showed that that the mutation rate in osteosarcomas is much higher in the non-coding regions (0.79 per Mb) than in coding regions (0.53 per Mb). Being a whole-genome sequencing dataset, the INOVA cohort lends itself as an independent dataset to be studied in-depth in future research projects. Hence there is much promise in the analysis and exploration of the whole genome for future research work in OS.

## Conclusion

Most publications that study drug response in Osteosarcoma focus on the exonic regions. However, most of the studies of OS have not been focusing on whole genome analysis with regard to treatment response. We hypothesized that genetic variation of the *host* may account for the wide variation seen in the response and toxicity related to chemotherapeutic agents. Our analysis approach was different from other studies that search for individual SNPs to confer significance. Using groups of SNPs for analyses has increased power in finding associations as well as increased robustness in statistical testing.

From our analyses, we found a list of intronic and intergenic hotspot regions common to both the TARGET and INOVA datasets that are significantly associated with outcome, providing insights into drug response mechanisms. Some of the genes with variants found in this study are linked with drug response at the pathway level (gene/pathway/biological processes level). Our results include variants in genes not previously linked with drug response, as well as novel SNPs and haplotypes in genes known to be linked with drug resistance. The targeted single SNP analysis of the DMET genes found variants significantly associated with both tumor necrosis outcome and survival.

We were able to validate the majority of the variants in our results using Sanger sequencing at the individual patient level. Identification and validation of such genetic markers that predict drug treatment response provide the basis for prospective evaluation of these candidate markers, and for future upfront treatment design based on individual genomic profiles.

## Additional files

Additional file 1: The steps, file formats and tools in the genomic data processing. (DOCX 148 kb)

Additional file 2: Detailed processing steps of Analysis 2 including the block diagram, filtering steps, PCA plot, equations of the generalized linear models. (DOCX 1860 kb)

Additional file 3: Details of the 231 haplotypes overlapping between the two datasets. (XLSX 83 kb)

Additional file 4: Details of the 281 variants significantly associated with tumor necrosis. (XLSX 114 kb)

Additional file 5: The confusion matrix and the summary of the generalized linear models. (DOCX 71 kb)

Additional file 6: Details of the Sanger Sequencing validation. (XLSX 88 kb)

## Abbreviations

3′ UTR: Three prime untranslated region
ADME: Absorption, metabolism, distribution, and elimination
ANOVA: Analysis of variance
CAM-DR: Cell adhesion-mediated drug resistance
COG: Children’s Oncology Group
DMET: Drug Metabolizing Enzymes and Transporter
FDR: False discovery rate
GLMs: Generalized linear models
GWAS: Genome wide association studies
haploblock: Haplotype block
LD: Linkage disequilibrium
MTX pathway: Methotrexate metabolic pathway
OS: Osteosarcoma
PCA: Principal component analysis
piR-Ls or pilRNA: piRNA-like
piRNA: Piwi-interacting RNA
SNPs: Single nucleotide polymorphisms
WGS: Whole genome sequencing
