# Supplementary material for: Genome sequencing analysis of blood cells identifies germline haplotypes strongly associated with drug resistance in osteosarcoma patients
Source: BMC Cancer. 2019 Apr 16;19:357. doi: 10.1186/s12885-019-5474-y (PMC6466653; doi:10.1186/s12885-019-5474-y)
Supplement: Supplementary file 1 — The steps, file formats and tools in the genomic data processing. (DOCX 148 kb) [file 12885_2019_5474_MOESM1_ESM.docx]

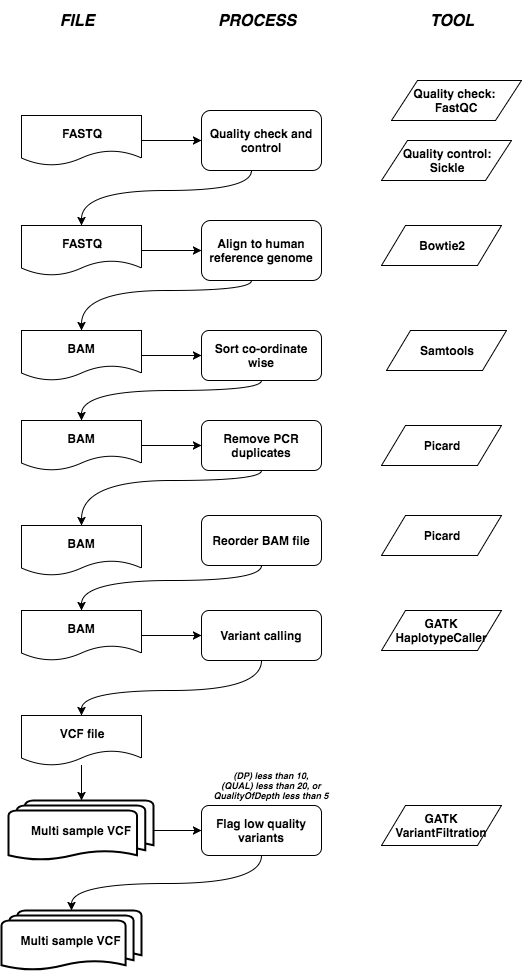


**Supplementary File 1. Shows the processing steps performed on the genomic data. Image format was taken from [31]. Raw sequencing FASTQ data [22] ranged from 50-140 GB each. Aligned reads were in the form of Binary alignment map (BAM) files [25]. The variant calls were in the form of Variant Call Format (VCF) file [32].**
