# Supplementary material for: Genome sequencing analysis of blood cells identifies germline haplotypes strongly associated with drug resistance in osteosarcoma patients
Source: BMC Cancer. 2019 Apr 16;19:357. doi: 10.1186/s12885-019-5474-y (PMC6466653; doi:10.1186/s12885-019-5474-y)
Supplement: Supplementary file 2 — Detailed processing steps of Analysis 2 including the block diagram, filtering steps, PCA plot, equations of the generalized linear models. (DOCX 1860 kb) [file 12885_2019_5474_MOESM2_ESM.docx]

# Supplementary File 2

## Detailed processing steps of Analysis 2 including the block diagram, filtering steps, PCA plot, equations of the generalized linear models

Out of 85 TARGET patients, only 44 had clinical data available on tumor necrosis (**Table 1(A)**). All the 15 INOVA patients had data available on tumor necrosis. Since the sample size would be small and there would not be enough power to obtain statistically significant results, we merged the two datasets for this analysis to get a total of 59 patients (referred to as the ‘TARGET+INOVA’ cohort).

After merging the two datasets, we got a multi-sample VCF file with about 26 million variants. We selected only those variants that had the “PASS” tag based on quality check, which gave us about 5.9 million variants. Out of this, we extracted variants in the DMET genes that gave us 36,504 variants.

Before start of analysis, we first tested the data for batch effects. We performed a principal component analysis (PCA) on variants in DMET genes (**Figure S2 (A)**) using the R platform (<https://cran.r-project.org/>). We found a clear batch effect due to the merging of the two datasets. We have accounted for this batch effect in our analysis.


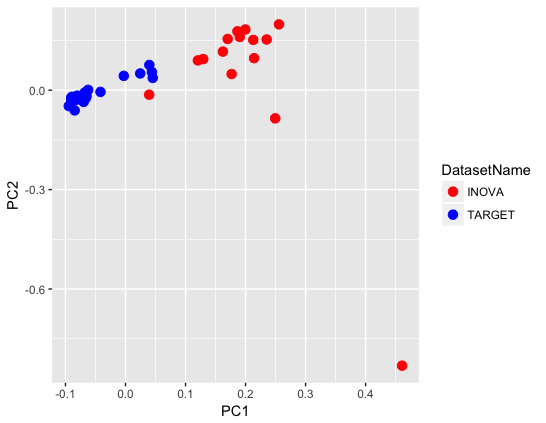


**Figure S2(A): Principal component analysis (PCA) showing clear separation between the datasets on the first two principal components**

We binned the data into 0 (indicating no variation) and 1 (indicating presence of variation) in R. 85% of this data was randomly set as training set and the rest 15 % was set as independent validation set using the caret package [35] in R (seed of 7 was used). In our dataset, about 60% of patients are ‘Poor responders’, 40% are ‘Good responders’. So the caret package automatically split the data into ‘poor responder’ and ‘good responders’ in this same proportion.

Several filters were applied on the training set so that only SNPs with the most variability were used for the analysis. If a SNP had reference or no call across all the samples, or had the same mutation across all the samples, there is no new information due to the lack of variability. This SNP was then excluded from analysis. Out of a total 36,504 SNPs, 6803 passed this filter. In addition to this, only SNPs that had a variance more than the median variance value were selected for analysis. After this filter was applied, we were left with 4543 SNPs for analysis.

For each of the 4543 variants, we created two generalized linear models (GLMs) with tumor necrosis as outcome variable: one specified with the variants and adjusting for the dataset to control for batch effect; and one specified without the variants (with only the dataset variable). We then used ANOVA to compare the two models to obtain a p-value based on chi-square distribution, shown in the equations shown below.

$$lmFit1 = glm\left( TumorNecrosisBin \sim DatasetName, family = \text{binomial} \right)$$

$$lmFit2 = glm\left( TumorNecrosisBin \sim{SNP}_{i}+ DatasetName, family = \text{binomial} \right) where i=1 to total number of SNPs$$

$$anovaOut = anova(lmFit1, lmFit2, test="Chisq")$$

This allowed us to perform logistic regression analysis to find those variants most associated with tumor necrosis outcome.

The p-values from this test were adjusted for multiple testing using the Benjamini Hochberg approach to control the false discovery rate (FDR) [36]. The significant variants (p value < 0.05) from this analysis were short listed for the next analysis step – we built a Random Forest predictive model with these significant variants with 20 fold cross validation (seed 260), and performed a prediction on the independent validation set. **Figure S2(B)** shows a block diagram representation of this methodology and **Figure S2(C)** shows the filtering steps.

**Figure S2 (B): Block diagram representation of the DMET analysis**


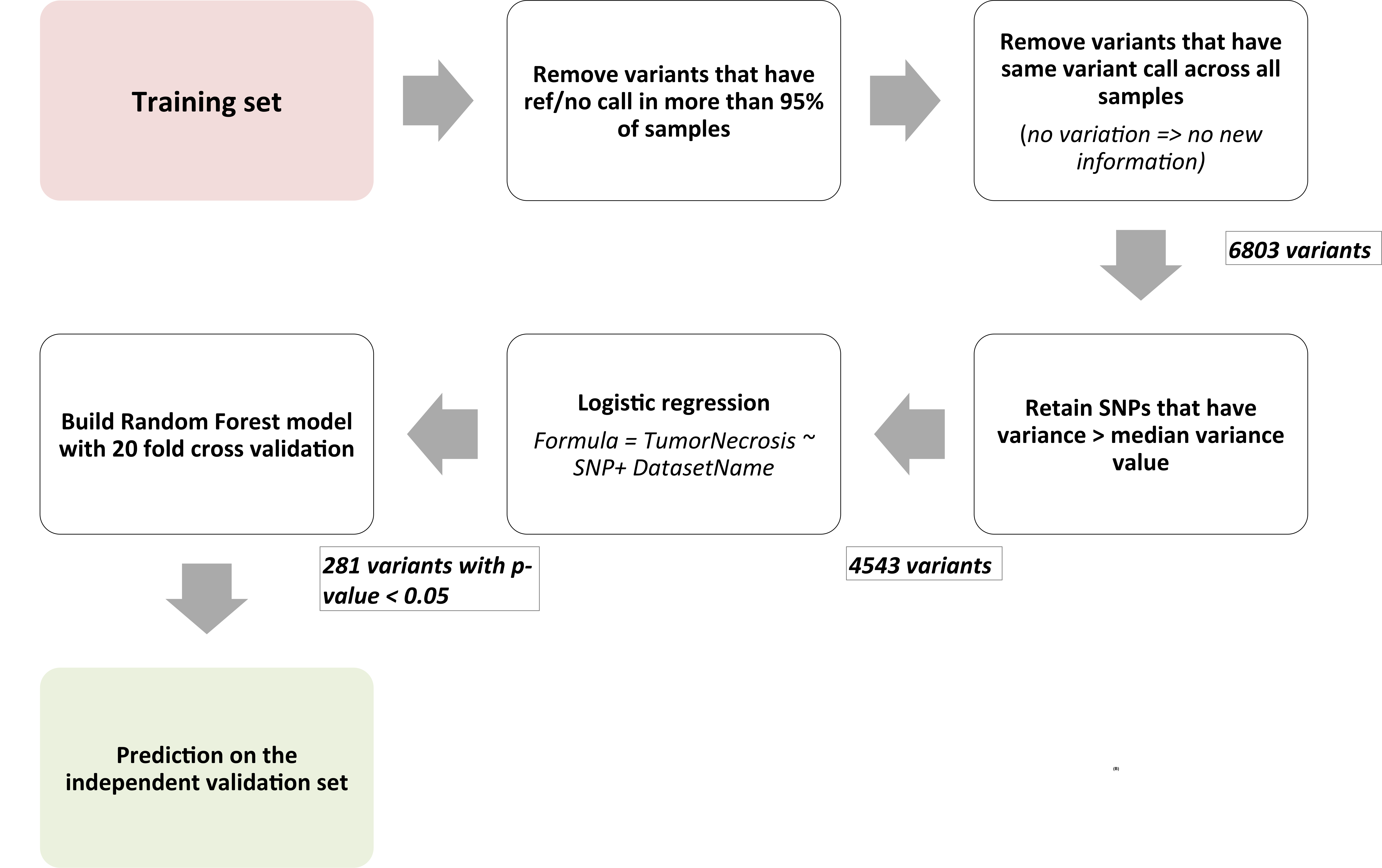


**Figure S2 (C): Filtering steps for the targeted DMET analysis**

The significant SNPs from this analysis were annotated using SnpEff [37] variant annotation tool. The annotations were used to sub-divide these SNPs based on their impact into high, moderate, low impact, and modifiers (SNPs non-coding regions).

Survival analysis was performed on these 281 significant SNPs to see their impact on overall survival. This analysis was performed in the R programming language using the survival package [38]. The association with survival was tested using the log rank test statistic [38], and Kaplan Meier survival curves [39] were also generated. We also looked up these results in published literature to see what results were already known.
