# Supplementary material for: Genome sequencing analysis of blood cells identifies germline haplotypes strongly associated with drug resistance in osteosarcoma patients
Source: BMC Cancer. 2019 Apr 16;19:357. doi: 10.1186/s12885-019-5474-y (PMC6466653; doi:10.1186/s12885-019-5474-y)
Supplement: Supplementary file 5 — The confusion matrix and the summary of the generalized linear models. (DOCX 71 kb) [file 12885_2019_5474_MOESM5_ESM.docx]

**Results of Random Forest model prediction on independent validation set**

|  | Reference | | |
| --- | --- | --- | --- |
| Prediction | **0 (No mutation)** | **1 (Mutation present)** | **Total** |
| 0 (No mutation) | 2 | 0 | 2 |
| 1 (Mutation Present) | 1 | 5 | 6 |
| Total | 3 | 5 | 8 |

**Table S5.1: Confusion matrix**

| **Model Statistics** | |
| --- | --- |
| Accuracy | 0.875 |
| 95% Confidence Interval | (0.4735, 0.9968) |
| No Information Rate | 0.625 |
| P-Value [Acc > NIR] | 0.135 |
| Kappa | 0.7143 |
| Mcnemar's Test P-Value | 1.000 |
| Sensitivity | 0.6667 |
| Specificity | 1 |
| Pos Pred Value | 1 |
| Neg Pred Value | 0.8333 |
| Prevalence | 0.375 |
| Detection Rate | 0.25 |
| Detection Prevalence | 0.25 |
| Balanced Accuracy | 0.8333 |
| Positive' Class | 0 |

**Table S5.2: Summary statistics of the Random Forest model**
